# Health Research and Knowledge Mobilization Priorities for Youth Health Equity in Canada: Protocol for a Multi-Methods Study

**DOI:** 10.2196/84782

**Published:** 2026-09-16

**Authors:** Carla T Hilario, Raissa Amany, Stacie Smith, Caroline Foster-Boucher, Priscilla N Boakye, Christine Cassidy, Egbe B Etowa, Olivia Stevenson, Bukola Salami, Josephine Pui-Hing Wong

**Affiliations:** 1 School of Nursing Faculty of Health and Social Development University of British Columbia-Okanagan Kelowna, BC Canada; 2 Young Canadians Roundtable on Health Toronto, ON Canada; 3 Faculty of Nursing Macewan University Edmonton, AB Canada; 4 Daphne Cockwell School of Nursing Toronto Metropolitan University Toronto, ON Canada; 5 School of Nursing Dalhousie University Halifax, NS Canada; 6 Department of Community Health Sciences University of Calgary Calgary, AB Canada

**Keywords:** youth, adolescents, health equity, social determiants, multi-methods, focus groups, group concept mapping

## Abstract

**Background:**

Health inequities have been a public health challenge for decades, exacerbated by disadvantages such as housing precarity, food insecurity, social disconnection, and discrimination, which are key social and structural health determinants. Young people who experience social disadvantages also experience a greater burden of health inequities. However, there has been limited research engaging youth in setting priorities for action and knowledge generation.

**Objective:**

Our study aims to develop a research and knowledge mobilization agenda for promoting health equity among Canada’s youth. The study will address important knowledge gaps through two study objectives: (1) to explore youth experiences of health equity and inequity and their perspectives on priority areas of need and (2) to collaboratively identify health equity priority areas for research and knowledge mobilization.

**Methods:**

The multi-methods study design is informed by the social ecological model and health equity principles. To address objective 1, a qualitative descriptive approach was used in phase 1 to elicit the perspectives of youth on their experiences related to health equity and inequity. Youth (aged 15-24 years) were recruited in 4 Canadian provinces, and data were collected using affinity-based focus groups, which will be analyzed using reflexive thematic analysis. To address objective 2, group concept mapping methods are being used in phase 2 to collaboratively identify health equity priority areas with knowledge users. Diverse knowledge user groups, including service providers, parents and caregivers, and youth participants from phase 1, are being engaged from across the research sites to participate in online synchronous group concept mapping sessions, which are informed by the focus group data. Integrated knowledge translation strategies are embedded in the research design to engage youth and relevant interest holders.

**Results:**

This project received funding from the Canadian Institutes of Health Research starting in July 2024. Ethics approvals from 5 participating universities were secured by October 2024. Data collection for phase 1 was conducted between November 2024 and April 2025. Data collection for phase 2 began in November 2025. Across the 2 phases, 113 participants have been engaged. Data analysis for both phases is underway, and results are expected to be published by March 2027.

**Conclusions:**

Youth perspectives on priority health needs and the historical and existing facilitators of and barriers to meeting these needs are needed to inform future research and action toward health equity. This research will contribute important knowledge about inclusive and meaningful engagement of systematically marginalized communities in the cogeneration of knowledge and research priorities.

**International Registered Report Identifier (IRRID):**

DERR1-10.2196/84782

## Introduction

### Background

Advancing equity is a key action area identified in Canada’s youth policy and the first State of Youth Report [1,2]. This action area is reflected in national research agendas such as the Canadian Institutes of Health Research (CIHR) Inspiring Healthy Futures report, which highlights the need for knowledge and action that put youth and families from diverse backgrounds at the center to reduce health inequities [3]. The Inspiring Healthy Futures report identifies the importance of collaboration, youth and family participation, and health equity in guiding collective action toward measurable improvement in the health and well-being of young people.

The basis for the goal of health equity is the growing body of evidence that links social and economic disadvantage to avoidable illness, disability, and premature death [4] and the recognition that these disadvantages can be addressed through social policies (such as those aimed at eliminating discrimination based on race, gender, disability, etc). There is mounting evidence indicating strong associations between social factors and health outcomes (eg, inequities based on racial identity [5-8]). Young people who experience social disadvantages due to structural determinants also experience greater burden of health inequities [9]. Chronic health conditions can increase health care needs that can exacerbate the impacts of these determinants [10]. This is important amid growing calls to address health equity and foreground youth perspectives to enhance the relevance and impact of health equity work [1].

The World Health Organization (WHO) Commission on Social Determinants of Health describes health inequities as “killing people on a grand scale” [11]. Health inequities are systemic differences in health that are caused by the uneven distribution of wealth, resources, and power in society; these differences in health are avoidable, unnecessary, and unjust [12]. Health inequities have been a public health challenge for decades, exacerbated by disadvantages such as housing precarity, food insecurity, social disconnection, and discrimination, which are key social and structural health determinants. According to the WHO World Report on Social Determinants of Health Equity, these inequities are most evident internationally in relation to economic inequality, structural discrimination, climate change, and digitalization [13].

In Canada, inequities in health and health care result from the intersecting impacts of social and structural determinants of health, including but not limited to income, racism, and colonialism [14]. For example, injuries and death are higher among young people in neighborhoods composed mostly of low-income, immigrant, and racialized populations [15]. It is also known that suicide rates are higher among First Nations, Inuit, and Métis youth in Canada [16,17]. Research suggests that youth who are disproportionately affected by health and social inequities are also more likely to be underrepresented in health promotion programs and report poorer access to care [3]. This evidence points to significant challenges in moving toward health equity, which refers to no youth being denied the possibility to be healthy due to belonging to a group that is economically and/or socially disadvantaged.

Action toward health equity requires responsiveness to the needs of groups who are at greater risk of poor health based on social conditions while also striving for optimal health for all people [18]. Equity work is grounded in a commitment to reduce disparities in health through addressing social determinants [19]. As a societal goal, health equity focuses attention on social determinants of health (SDoH) to monitor and address health inequities at the population level [20]. Central to discussions of health equity is the question of what constitutes evidence that health equity has been advanced and how the principle can be operationalized for measurement. There is a noted need for innovation in applying a health equity lens to a specific scope of work, communicating about health equity in ways that can be understood within and beyond public health, and gathering support for integrating health equity amid competing priorities and maintaining commitment and resources [5].

Internationally, specific gaps in policy and action include addressing economic inequality by funding universal public services, expanding coverage of universal social protection systems for all, and evaluating the commercial determinants of health [13]. To address structural discrimination, actions include identifying discrimination embedded in policies, laws, institutions, and social norms; redressing the impacts of colonization; and safeguarding the SDoH equity during migration, emergencies, and conflict. Importantly, the WHO recommends new governance approaches to bring about change, including supporting community engagement in local policy processes and creating the enabling conditions that maximize the capabilities of inclusive civil society to address the SDoH equity. This study responds to these recommendations by creating enabling conditions that support the engagement of youth in addressing the SDoH equity.

Definitions of health equity vary, and although youth-focused organizations are foregrounding policies and strategic plans in terms of health equity and SDoH, there is a need to identify priority areas toward translating these plans into meaningful action. Furthermore, research on youth perspectives on and experiences of health equity and inequity need to inform priority-setting research and initiatives. Importantly, the mobilization of knowledge into action must be in collaboration with youth and predicated on their rights to express their perspectives and engage in issues affecting their health [21]. Youth perspectives provide critical information about health needs, historical and persistent facilitators and barriers, and dynamic understandings of health equity, which are all deeply linked to sociopolitical contexts. However, the perspectives of young people are often missing in prevailing approaches to identifying and setting priorities for action, knowledge mobilization, and research.

Identifying health equity priorities requires meaningful engagement with young people living in that context as well as with groups who strive to recognize and address the health needs of youth. In Canada, youth-led priority setting is currently lacking in existing health equity research, particularly in work focusing on knowledge mobilization. Furthermore, there is limited cross-provincial work in this area, which would foster critical dialogues based on youth-identified priorities.

### Study Objectives

To address these knowledge gaps, our study objectives are (1) to elicit the perspectives of youth on their experiences related to health equity and inequity, including priority areas of need; and (2) to collaboratively identify health equity priority areas with diverse youth, as well as parents, caregivers, and service providers. The study uses affinity-based focus groups and group concept mapping (GCM) methods, which are designed for group engagement, dialogue, and consensus building and are well suited to address the research gaps. This novel project will generate knowledge on experiences of health equity and inequity among youth in Canada and apply those insights in collaboratively identifying priority areas in health equity research and knowledge mobilization.

The conceptual framework used in designing the study draws on the concept of health equity as “the principle underlying a commitment to reduce—and, ultimately, eliminate—disparities in health and in its determinants, including social determinants” [19]. The study is guided by social ecological theory, which draws attention to multiple, nested levels of influence and determinants that interact to shape health outcomes at various levels [22,23]. A social ecological model of health framework will inform the conceptualization and presentation of multiple levels of influence and actions in relation to SDoH during data analysis, data interpretation, and knowledge mobilization (Figure 1). In addition, the study design is informed by critical social theoretical perspectives that position health inequities within power dynamics that are embedded in economic, political, and historical contexts and attend to health outcomes as well as access to health care and health-promoting resources for health at a collective level [24,25].

**Figure 1:**
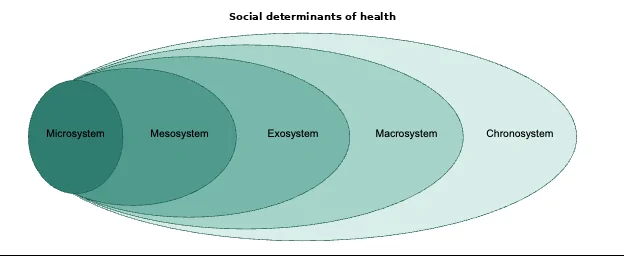
Study conceptual framework.

## Methods

### Overview

This multi-methods study has a 2-phase design, with knowledge mobilization integrated into the second phase and in end-of-grant activities. The first phase of the study used a qualitative descriptive approach and reflexive thematic analysis (RTA). The second phase used GCM, a mixed methods approach combining qualitative and quantitative research methods. The data gathered from phase 1 were used to inform phase 2 (Figure 2). The specific methods are detailed within each phase.

**Figure 2:**
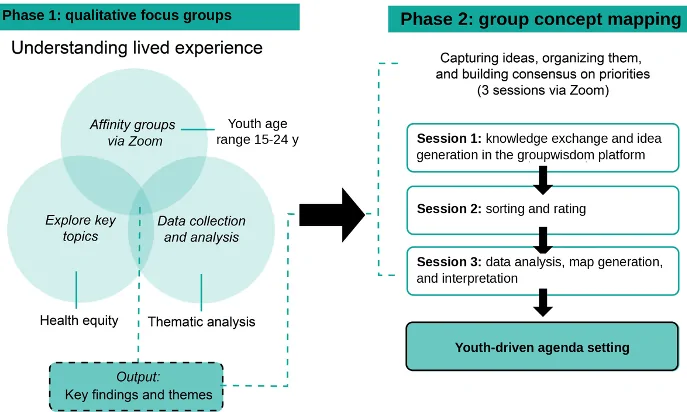
Study phases and research activities.

### Phase 1 (Objective 1): To Explore Youth Experiences of Health Equity and Inequity

#### Methodology

To address the first objective, a qualitative descriptive approach [26] was used to elicit the perspectives of youth on their experiences related to health equity and inequity, including priority areas of need. This phase foregrounded the perspectives of diverse minoritized youth living in 4 Canadian provinces—British Columbia, Alberta, Ontario, and Nova Scotia—to gain a unique understanding of contextual determinants of health equity and inequity within and across these contexts nationwide. The feasibility of this design was enhanced by the national research team’s existing networks. An RTA approach will be used to guide data analysis. This phase will be conducted and reported in accordance with the COREQ (Consolidated Criteria for Reporting Qualitative Research) guidelines to ensure methodological transparency [27].

#### Sampling and Recruitment

The study sample in this phase were youth aged 15 to 24 years and living in British Columbia, Alberta, Ontario, and Nova Scotia, which are provinces where the research team members are based. The target sample size was 40 to 60 participants across the research sites (10-15 youth per site). Consistent with previous qualitative studies with youth [28] and drawing on the concept of information power [29,30], this sample size was anticipated to generate contextually rich data through dialogue and sharing. Maximum variation purposive sampling was used to recruit a diverse group of youth to enhance representation across dimensions of identity likely to shape experiences of health equity and inequity, including gender identity, belonging to a racialized group, newcomer status, disability, and geographic location. The recruitment strategies were determined by the research team, which includes youth partners, to ensure that the sample captured a range of lived experiences and perspectives relevant to identifying health equity priority areas. These strategies included targeted recruitment efforts through youth-led and youth-focused organizations, including the Young Canadians Roundtable on Health, and through place-based recruitment (eg, flyers at community centers, libraries, etc) to engage and recruit youth of diverse socioeconomic; ethnic and racial; and gender-diverse two-spirit, lesbian, gay, bisexual, transgender, and queer identities. Advertisements on social media (eg, Instagram) and through youth-focused organizations were also used in recruitment. In addition, snowball sampling was used to identify other prospective participants from those who had similar characteristics (eg, being a newcomer).

#### Data Collection: Affinity Focus Group Discussions

Prior to participation, youth reviewed informed consent documents and provided consent using a university-hosted Qualtrics survey (Qualtrics International Inc). Participants were reminded of their voluntary and ongoing consent throughout all stages of the research process. Participants also completed a brief self-identification survey to provide information on the diversity of the study sample. The information was used to guide the organization of affinity focus groups. To gather this information, we used the current self-identification questionnaire used by the CIHR. The CIHR self-identification questionnaire covers 8 dimensions of identity: age, gender identity, sexual orientation, Indigenous identity, visible minority identity, population group, disability, and language [31].

The study drew on the concept of *affinity groups* to guide the design and coordination of the focus groups. Affinity groups are defined as groups that bring together individuals sharing common identities, backgrounds, or interests, such as women or Indigenous, Black, and people of color, to enhance a sense of inclusion and support. Several groups were predetermined based on the research team’s understanding of equity and inequity and prior relevant research [32-36]. The affinity group approach enhances psychological safety and courage by creating groups with typically greater commonalities in experience rather than a widely diverse group that may not share the same experiences of inequities; based on our team’s experience, this approach also helps mitigate stereotypes. These groupings included Indigenous youth; Black youth; racialized youth; two-spirit, lesbian, gay, bisexual, transgender, and queer youth; youth with and without disabilities; newcomers, refugees, and immigrants; and women-identifying youth. Participants were asked for suggestions on affinity groupings not yet included in the study. Following the consent process, participants were asked to self-select into affinity groups based on their identities and experiences. To make space and ensure safety for intersecting identities, participants could select more than one group. A list of provincial mental health resources for each of the 4 individual provinces was provided to each participant in advance of the focus groups.

Each focus group was facilitated by a research team member who self-identified with that affinity identity. Focus groups were 2 hours in length and facilitated using an interview guide including open-ended questions on the topic of health equity (Multimedia Appendix 1). The team planned to conduct 8 to 10 focus groups online via Zoom (Zoom Video Communications) across the 4 study sites. This number of focus groups ensured sufficient depth and richness in the qualitative data [29,30]. Each focus group included a target group size of 5 to 8 youth, which was an ideal size given the complexity of the topic, number of questions to be covered, and study objective of understanding health equity [37].

Focus group transcripts were automatically generated using a university-hosted Zoom license and its transcription feature. Each transcript will be checked against the audio recordings by 2 individual research team members to ensure accuracy and completeness. The NVivo (version 12 Plus; Lumivero) research software will be used to organize the transcribed interview data. Research notes tracked in a field notebook will inform data analysis.

#### RTA

RTA will be used to identify themes that capture a core idea or meaning about the data and provide meaning-based interpretation of those themes [38,39]. In this study, we locate our use of RTA theoretically within critical social theories to examine who benefits from power; who is marginalized; and how marginalization shapes people as well as structures, institutions, and practices [40]. We also draw on Black feminist intersectionality theory to inform our understanding of health equity and the ways in which people can be simultaneously oppressed and privileged based on varying aspects of their identity and help situate our identities, values, and disciplinary backgrounds as individual researchers and a collective team in relation to the study. As a collective team, we are guided by principles of social justice, which inform this study’s focus on health equity and SDoH and our approach to the analysis.

The data analysis process draws on steps commonly used in RTA, including (1) repeated readings of the transcribed focus groups; (2) generating initial broad-based codes inductively derived from the readings of the data; (3) developing a coding tree to guide the coding of the transcripts; (4) identifying themes based on the coding; (5) reviewing, describing, and naming the themes; and (6) producing a report of the themes supported by verbatim excerpts that will be informed by the study conceptual framework. Analytic logic will be tracked using an audit trail. We will follow an iterative team-based data analysis approach to integrate collectively generated insights and facilitate consensus. While RTA does not explicitly engage with qualitative research quality criteria such as confirmability, dependability, or credibility, we apply RTA strategies for enhancing quality, including reflexive journaling, gaining insights from the research team, allowing time for analytic insights to fully develop, naming themes carefully, and maintaining an audit trail [41]. In the analysis, we will also situate the data within the wider context to enable audiences to enhance the transferability or relevance of the study to other settings and contexts [41].

### Phase 2 (Objective 2): To Collaboratively Identify Health Equity Priority Areas for Knowledge Mobilization and Research

#### Methodology

To address objective 2, GCM will be used to collaboratively identify health equity priority areas with knowledge users drawing on youth experiences of equity and inequity. GCM integrates quantitative and qualitative research methods to examine participant-generated concepts using a series of visual representations, including point maps, cluster maps, cluster rating maps, pattern matches, and scatterplots [42]. The methodology provides a structured, rigorous process for engaging a diverse group in aggregating, interpreting, and contextualizing participant-generated insights (qualitative data), as well as how they are associated and the degree of relevance and importance (quantitative data). GCM is well suited for exploring lived experience in participatory health research [43] and identifying future strategic planning [44].

Informed by the study conceptual framework, which draws on the social ecological model and health equity principles, data generated in phase 1 will be used to inform phase 2 research activities. We will apply GCM methods to facilitate the individual generation of insights about health equity, which will then be analyzed using quantitative statistical methods to determine systematic patterns [45]. The goal of the GCM sessions will be to codetermine priorities of health equity knowledge mobilization and emerging research foci.

The GCM sessions will be held synchronously online via University of British Columbia (UBC)–hosted Zoom videoconferences and engage participants from across Canada. A target sample of 60 participants will be recruited to ensure adequate power for the quantitative GCM analysis based on existing literature [43,46,47]. Purposive sampling and snowball methods will be used to invite youth, parents and caregivers, service providers and leaders from youth-focused organizations, community advocates, interdisciplinary researchers, and knowledge users who play key roles in the lives of young people. There will be targeted outreach to organizations and networks serving equity-deserving youth, particularly in British Columbia, Alberta, Ontario, and Nova Scotia. The Concept Systems groupwisdom software will be used to support engagement of participants and facilitate analysis of the data [48]. The GCM activities will be conducted over 3 online sessions, which are described in the following sections.

#### Session 1: Knowledge Exchange and Idea Generation

In this step, the research team prepares and presents data from phase 1 on youth participants’ experiences of and perspectives on health equity and inequity. Participants are invited to discuss gaps and issues in health equity for youth, approaches to actively involving youth in efforts to address health equity, and elements of effective strategies that promote health equity. Participant insights further support our team in assigning meaning to the results from phase 1. The team and participants drew on emerging insights from the focus group discussions to collectively outline knowledge mobilization priorities and produce innovative research questions that can foster action toward health equity in Canada. After the discussion, the group is presented with the following focus prompt: “To achieve health equity for youth in Canada, action and research is needed to...” This prompt functions as a guide to help participants think broadly and work individually to generate as many ideas as possible. Participants are asked to generate each idea as a single short sentence and enter it directly into the groupwisdom platform. All entries are automatically collected as anonymous statements. Nominal group methods are used to capture ideas from the participants in a sequential order [49]. Before the next activity, the research team reviews all responses from participants to remove duplicate ideas and generate a final list for rating in session 2. The research team then collates generated ideas, and codes are assigned to each statement to organize the ideas. The final set of statements is uploaded to groupwisdom, where they are randomized, assigned a number, and exported to create individual statements.

#### Session 2: Sorting and Rating

In this session, the final list of participant-generated statements is shared with participants, who are asked to read each statement and group them into piles based on their perceived similarity. Participants then designate a name for each grouping and assign ratings to the ideas based on three criteria: (1) the importance of each item on a scale from 1 to 4, with 4 being the most important; (2) the current presence of that idea on a scale from 1 to 4, with 4 being the most present; and (3) the feasibility of each item on a scale from 1 to 4, with 4 being the most feasible. Participants enter their sorting and rating into the groupwisdom platform for analysis and generation of concept maps. New participants are recruited if needed to ensure a sufficient sample. The sorting and rating data are reviewed by the research team for analysis and interpretation prior to session 3.

#### Session 3: Data Analysis, Map Generation, and Interpretation

Using the groupwisdom software, the sorted data will be analyzed using multidimensional scaling and hierarchical cluster analysis. Multidimensional scaling will be used to generate point maps in which the sorting data are represented as points on 2D maps. These point maps highlight the similarities of items based on aggregate sorting data. Hierarchical cluster analysis will be used to generate 3D cluster maps, which will show the aggregate data from the sorting procedure to group collections of individual items and use the data from rating procedures to demonstrate the relative importance of these themes. These domains can also be reviewed and interpreted based on their 3D representations of importance indicated by participants. Boundaries will be drawn around sorted data points to represent conceptual groupings. A stress value range from 0 to 1 will be generated to indicate the goodness of fit of the mapped statements to the sorted data.

The participants will be invited to contribute to the interpretation of the concept maps; identify interconnections between issues; and outline priority areas for knowledge mobilization and novel questions to drive future research related to health equity, which is a component of the knowledge mobilization plan for the study. In this activity, the research team will present a set of concept maps with varying clusters and engage participants in discussion to determine which maps are most reflective of their ideas and priorities. Once the cluster map interpretation process is completed and a collective decision is made on the most appropriate cluster map, participants will be engaged in discussion to identify concrete next steps toward addressing the priority health equity needs of youth through knowledge mobilization and research.

### Ethical Considerations

The study protocols of the current study have been approved by the research ethics boards of all participating institutions: UBC (H24-00786), Dalhousie University (2024-7364), MacEwan University (102,350), Toronto Metropolitan University (2024-309), and University of Calgary (H24-00786). Written informed consent was required from all participants before taking part in the study. Participants were instructed to use pseudonyms during data collection sessions. Raw audio data will be permanently deleted after the transcriptions are verified. Transcripts will be further deidentified during transcription. Information collected for distributing participant compensation was stored separately from the research data and password protected. All documents were stored in research folders on the UBC Microsoft OneDrive service, with access restricted to key research team members. Participants were offered CAD $40 (CAD $1=US $0.72 as of August 13, 2026) in the form of an electronic gift card for taking part in a focus group. Participants of GCM sessions received a CAD $50 e-gift card for each session attended up to a maximum of CAD $150 per participant.

### Knowledge Mobilization

Knowledge translation and exchange is embedded in our research design. The research team includes youth leaders who were involved in the conceptualization and design of the study and as principal investigators on the project. Youth are also engaged as research trainees in the project and will be engaged in focus group qualitative data interpretation and theme development and in the analysis and interpretation of the concept maps. Aligned with the knowledge translation planning primer, we will use GCM as an integrated knowledge translation strategy to (1) identify and translate research findings into key messages; (2) engage end users; and (3) develop tailored outputs, including a research agenda that will be shared and tailored for different audience groups. Knowledge mobilization outputs will include social media posts and infographics targeted for youth, brief lay reports and webinars streamed through live social media broadcasts tailored for parents and caregivers and youth-serving organizations, and scientific presentations and academic publications for academic audiences. In assisting with the creation of knowledge mobilization outputs, youth research trainees will provide a youth perspective on the framing of key messages.

## Results

In this study, we apply multiple methods to explore youth experiences of health equity and inequity and collaboratively identify health equity priority areas for knowledge mobilization and research drawing on youth experiences of equity and inequity. Our research team includes representatives of a youth-led national organization as principal knowledge users. This project was funded in July 2024, and we secured research ethics board approvals across 5 Canadian universities by October 2024. Recruitment and data collection for phase 1 were conducted between November 2024 and April 2025. During this period, we engaged with community partners and collaborators to refine recruitment strategies. Data collection for phase 2 began in November 2025. As of June 2026, we had successfully engaged 48 youth across Canada to take part in the phase 1 affinity focus groups and 65 participants to take part in the phase 2 GCM sessions. Across the 2 phases, 113 participants have been engaged. Data analysis is underway; 2 peer-reviewed manuscripts are expected to be submitted for publication by March 2027.

## Discussion

### Expected Findings

The overall goal of our study is to advance youth health equity in Canada by identifying and prioritizing knowledge mobilization and research foci that will advance equity-oriented health services, health system change, and long-term sustained action toward improving youth health outcomes. According to the most recent United Nations Children’s Fund report on child well-being, Canada “barely received a passing grade” on child and youth health, with Canadian youth ranking 19th out of 36 comparable countries on overall health and well-being [50]. The report found greater health risks among youth marginalized by income, indigeneity, race, gender, and disability, along with other SDoH. A recent study assessed Canada’s success in reducing socioeconomic and health inequities in 7 areas to address SDoH, including investments in sexual and reproductive health and family planning, early learning and childcare, education, and universal health care, as well as investments to reduce child poverty, ensure sustainable economic development, and control health hazards [51]. The study found that Canada’s successes have been in reducing socioeconomic inequalities in early learning and childcare and reproductive health, and the least successful outcomes were found in relation to health hazard control and child poverty.

Drawing on our previous research on social determinants of youth health, health services [32,52-55], and minoritized groups [33,56-60], the study recognizes the diversity of youth in Canada and the importance of understanding the unique social and structural conditions that shape their health and well-being. In this vein, the study aims to intentionally engage diverse youth, including those from groups that may be underrepresented in research, programs, and policy and especially those who are minoritized due to SDoH. Importantly, this research engages youth in priority setting, which is currently lacking in extant health equity research, particularly in work focusing on knowledge mobilization.

This study centers youth health, voices, and experiences and embeds equity, diversity, and inclusion into the project design. Insights from youth perspectives will inform broader discussions with youth, parents, service providers, and others engaged in youth health and, through a structured approach, inform the generation of ideas related to health equity priority areas, which will be rated and analyzed to generate context-relevant recommendations. The expected outcomes of this study are (1) advancement of inclusive research methodology in engaging systematically marginalized and equity-deserving youth, (2) mobilization of cross-sectoral stakeholders to engage in committed research collaboration, (3) inclusive and meaningful engagement of racialized and systematically marginalized communities in cogeneration of knowledge and research priorities, (4) effective knowledge mobilization using data visualizations, and (5) trust building and leadership sharing in health research. The study aligns with national research strategic plans in Canada and its core focus on empowered youth [61]. The knowledge generated by the study can be applied in community-based and public health responses to strengthen efforts in addressing health inequities that affect the lives of young people in Canada and can inform the development and/or adaptation of health equity interventions that are context relevant and evidence based.

### Dissemination of Findings

The dissemination of findings in our study reflects the complexity of youth health equity by using approaches that are accessible and inclusive to the needs of various audiences. Consistent with the methods used in the study design, dissemination will prioritize communication strategies that focus on engaging multiple channels [62]. As such, the findings from this work will be tailored to its various interest holders, including youth, families and caregivers, youth-serving organizations, health care systems, and academic audiences.

Knowledge mobilization is embedded throughout this study, particularly within the GCM process, wherein participants are invited to reflect on data from the focus groups and share these insights within their own communities. This process supported early dissemination and interpretation. Dissemination will continue through targeted activities. These activities will take place across multiple audience levels and formats. At a public awareness and engagement level, findings will be shared through online platforms and social media to increase accessibility and reach, with the intention of engaging youth, caregivers, and organizations. Participants who expressed interest in receiving study results will also be provided with plain-language summaries, which may also support further sharing within their personal and professional networks. Professional engagement will include the development of brief lay reports and webinars designed for community- and practice-based audiences. Additionally, academic dissemination will include conference presentations, academic publications, and a research agenda, ensuring that findings contribute to broader conversations on youth health equity. Importantly, all dissemination activities will continue to be informed through youth engagement so that messaging remains relevant and impactful. This approach reinforces the study’s commitment to not only sharing knowledge but also supporting its application in ways that reflect the priorities and experiences of youth.

### Study Strengths and Limitations

Notable strengths include the multi-methods, multiphase design. By integrating qualitative descriptive methods with GCM, this study offers a multi-methods approach to engaging with youth and understanding their experiences of health equity and inequities. Youth engagement was further supported through intentional strategies to promote safety, such as self-selection into affinity groups and the prioritization of participation among youth from equity-deserving groups. Where possible, affinity focus groups were facilitated by individuals who shared similar identities. Additionally, cross-provincial engagement strengthened the relevance of this work across the Canadian context, supporting the transferability of findings. However, engagement from one site was limited, and the study only focused on 4 provinces in Canada. As a result, this study may not fully capture the perspectives and experiences of all youth in Canada, particularly those in the Atlantic provinces. Similarly, due to the virtual nature of data collection, individuals without reliable access to devices or internet connection may have been unable to participate, potentially limiting representation. Challenges were also encountered in online recruitment and verification processes. To address this, several measures were taken to reduce the risk of falsely identifying participants, including identity verification at the start of focus group sessions and advance screening procedures for GCM sessions. Despite these limitations, this study is driven by the perspectives of youth and those who serve them across Canada.

### Conclusions

In conclusion, it is essential that youth health equity is addressed in ways that are meaningful and responsive to the lived experiences of youth. Doing so can promote lifelong health and expand access to care, enabling individuals to experience fewer inequities across the life course. Insights from this study will inform future research agendas and support the development of health system changes that better meet the needs of youth both now and as they transition into adulthood. Ultimately, this work reinforces the recognition that achieving health equity requires ongoing engagement with those most affected, ensuring that approaches are informed by youth and lead to meaningful change.

## Data Availability

The datasets generated and analyzed during this study are available from the corresponding author on reasonable request.

## Appendix

Multimedia Appendix 1 Focus group guide.

Multimedia Appendix 2 Peer Review Report by Catalyst Grant: Healthy Youth Review Committee, Canadian Institutes of Health Research (CIHR) (Committee Member 1).

Multimedia Appendix 3 Peer Review Report by Catalyst Grant: Healthy Youth Review Committee, Canadian Institutes of Health Research (CIHR) (Committee Member 2).

Multimedia Appendix 4 Peer Review Report by Catalyst Grant: Healthy Youth Review Committee, Canadian Institutes of Health Research (CIHR) (Committee Member 3).

## Abbreviations

CIHR: Canadian Institutes of Health Research
COREQ: Consolidated Criteria for Reporting Qualitative Research
GCM: group concept mapping
RTA: reflexive thematic analysis
SDoH: social determinants of health
UBC: University of British Columbia
WHO: World Health Organization
