# Supplementary material for: Health Research and Knowledge Mobilization Priorities for Youth Health Equity in Canada: Protocol for a Multi-Methods Study
Source: JMIR Res Protoc. 2026 Sep 16;15:e84782. doi: 10.2196/84782 (PMC13628041; doi:10.2196/84782)
Supplement: Supplementary file 1 [file resprot_v15i1e84782_app1.pdf]

## Phase One – Focus Group Guide

In this phase, we will conduct focus groups to explore youth experiences of health in/equity and their perspectives on priority areas of need, historical/existing facilitators, and barriers for them in meeting those needs, and opportunities for increasing access and equity (research objective 1).

Introduction:

- Welcome and thank participants for joining the study.
- Briefly review the purpose of the study and focus of the discussion- To explore youth experiences of health in/equity and their perspectives on areas of need and opportunities for increasing access and equity.
- Review consent form information: Confidentiality is expected but cannot be guaranteed, right to leave or not answer, respectful engagement, etc.
- Emphasize the importance of youth perspectives in shaping initiatives to address health disparities and promote equity.
- Refer to the mental health resource list that the participants can use if they need support during or after the focus group (Youth mental health resources- BC, AB, ON, and NS based on where the participant is located)
- Respond to any participant questions.
- Facilitate brief icebreaker.

Focus group questions:

1. Health inequities mean that some people are healthier than others because of differences in money, resources, and power in society. Even though everyone should have a fair chance to be healthy, some groups of young people are more likely to get sick because they don't have as much money, face racism, have disabilities, or experience other social differences. These young people may also have more difficulty using health care services and accessing programs to improve their health.
    - Do you have any questions about this?
    - Could you please share your thoughts on what health equity or health inequity means to you as a youth or young adult?

2. From your perspective, what are some of the biggest health-related challenges facing youth today?

3. Can you describe your experiences or observations of differences in *health outcomes* for different youth or communities of youth?
    - How about barriers or challenges to *accessing* healthcare or health-related resources- Have you experienced or witnessed any barriers to accessing hospitals, services in the community, or online materials?

4. What factors do you believe contribute to these differences or inequities? Why do you think so?
5. Have you experienced any facilitators or support systems that have helped you or other youth address your/their health needs?
6. From your perspective, what do you think are the most important or urgent health equity issues that researchers and policymakers should focus on?
    - Are there any specific topics or areas related to health equity that you feel should receive more attention or need more awareness? Why do you think so?
7. How do you think young people can be more actively involved in efforts to address health equity?
8. Can you suggest any strategies or approaches for increasing access to healthcare and promoting equity among youth populations?
9. Is there anything else you would like to share that we haven't talked about today?

Closing:

- Thank participants for their valuable insights and for contributing to the discussion. Reiterate the importance of their voices in promoting health equity in their communities.
- Remind participants of confidentiality- Not sharing what was discussed in the focus group with others.
