# Supplementary material for: Health Research and Knowledge Mobilization Priorities for Youth Health Equity in Canada: Protocol for a Multi-Methods Study
Source: JMIR Res Protoc. 2026 Sep 16;15:e84782. doi: 10.2196/84782 (PMC13628041; doi:10.2196/84782)
Supplement: Supplementary file 2 [file resprot_v15i1e84782_app2.pdf]

**Review Type/Type d'évaluation:**	Committee Member 1/Membre de comité 1
**Name of Applicant/Nom du chercheur:**	Hilario, Carla Theresa
**Application No./Numéro de demande:**	513361
**Agency/Agence:**	CIHR/IRSC
**Competition/Concours:**	2023-11-08 Catalyst Grant: Healthy Youth/Subvention catalyseur : Jeunes en santé
**Committee/Comité:**	Catalyst Grant : Healthy Youth/Subvention catalyseur : Jeunes en santé
**Title/Titre:**	Identifying health research and knowledge mobilization priorities for promoting health equity in Canada's youth

Assessment/Évaluation:

Summary:
The purpose of this proposed research is to develop a knowledge generation and knowledge mobilization agenda for promoting health equity in Canada’s youth. The study will address important knowledge gaps through two study objectives: (1) to explore youth experiences of health in/equity and their perspectives on priority areas of need; (2) to co-identify health equity priority areas for research and KM. To meet objective 1 a qualitative descriptive approach will be used to elicit the perspectives of youth on their experiences related to health in/equity, including priority areas of need. This phase will foreground the perspectives of racialized minority youth of diverse gender and sexuality. They will recruit up to 60 youth from 15 to 24 years of age across four research sites using place-based outreach approaches to engage equity-deserving youth of diverse socioeconomic, ethnic/racial, and gender diverse 2SLGBTQ+ identities. They will conduct 8-10 online focus groups via Zoom with 6-8 youth per focus group across the four sites. To meet objective 2 group concept mapping (GCM) will be used to collaboratively identify health equity priority areas with knowledge users, drawing on youth experiences of in/equity. They will recruit 60 participants from across Canada to participate in online synchronous GCM sessions. The qualitative data to meet objective 1 will be used to inform the GCM workshop discussions with a view to co-develop novel research questions that are significant for health equity.

Strengths:
This is an under-researched topic with a population that has not been sufficiently engaged in the past.
The focus on developing research questions is novel and has the potential to support strong future research in this area.
The analysis approach for the focus groups is well described.
The group concept mapping is well-described from a methodological point of view.
The team is strong and includes early career researchers as well as youth in leadership roles. The team is well-positioned to carry out this research.

Weaknesses
It is unclear what specific themes will be covered in the focus groups, i.e. how is the topic of health in (equity) going to be brought to the groups? Inequities are not always easy to explain and some concrete explanations will be needed to engage the youth, especially those who are not post-secondary students.
The same challenge applies to the group concept mapping. Details are needed explaining how the concepts will be presented to participants. It is stated that the qualitative data from the focus groups will inform the concepts, but given there is not a lot of specificity in terms of how heath equity will be introduced to the focus group participants, further clarity is needed here.
The purposive sampling approach needs clarity. The statement about the types of organizations where youth will be sought is vague and it isn't until the letters of support that some specificity is provided. Simply stating that LGBTQ2S+, Black mentorship or other organizations will be involved in recruitment would have added needed clarity.
