# Supplementary material for: Health Research and Knowledge Mobilization Priorities for Youth Health Equity in Canada: Protocol for a Multi-Methods Study
Source: JMIR Res Protoc. 2026 Sep 16;15:e84782. doi: 10.2196/84782 (PMC13628041; doi:10.2196/84782)
Supplement: Supplementary file 3 [file resprot_v15i1e84782_app3.pdf]

**Review Type/Type d'évaluation:**	Committee Member 2/Membre de comité 2
**Name of Applicant/Nom du chercheur:**	Hilario, Carla Theresa
**Application No./Numéro de demande:**	513361
**Agency/Agence:**	CIHR/IRSC
**Competition/Concours:**	2023-11-08 Catalyst Grant: Healthy Youth/Subvention catalyseur : Jeunes en santé
**Committee/Comité:**	Catalyst Grant : Healthy Youth/Subvention catalyseur : Jeunes en santé
**Title/Titre:**	Identifying health research and knowledge mobilization priorities for promoting health equity in Canada's youth

---

Assessment/Évaluation:

<u>Identifying health research and knowledge mobilization priorities for promoting health equity in Canada's youth</u>

The applicants make the case that youth perspectives on issues of health equity have not been available or considered.

The purpose of this proposed research is to develop a knowledge generation and knowledge mobilization (KM) agenda for promoting health equity in Canada’s youth. The study will address important knowledge gaps through two study objectives: (1) to explore youth experiences of health in/equity and their perspectives on priority areas of need; (2) to co-identify health equity priority areas for research and KM

First objective, they will use a qualitative descriptive approach to elicit the perspectives of youth on their experiences related to health in/equity, including priority areas of need. Will involve perspectives of racialized minority youth of diverse gender and sexuality and are living in British Columbia, Alberta, Ontario, and Nova Scotia

They will recruit up to 60 youth from 15 to 24 years of age across the research sites (10-15 youth per site).

Objective 2 is group concept mapping (GCM) to collaboratively identify health equity priority areas with knowledge users, drawing on youth experiences of in/equity

They will recruit 60 participants from across Canada to participate in online synchronous GCM sessions. This sample size will ensure adequate power (n 40) for the GCM process

The GroupwisdomTM web platform (Concept Systems Inc.) will be used to engage participants and analyze the data in five steps.

Strengths

Strong team with expertise

Well written grant in terms of justifying the need and the problem, explaining/justifying methods, trying to be representative geographically

Areas for improvement

**Review Type/Type d'évaluation:**	Committee Member 2/Membre de comité 2
**Name of Applicant/Nom du chercheur:**	Hilario, Carla Theresa
**Application No./Numéro de demande:**	513361
**Agency/Agence:**	CIHR/IRSC
**Competition/Concours:**	2023-11-08 Catalyst Grant: Healthy Youth/Subvention catalyseur : Jeunes en santé
**Committee/Comité:**	Catalyst Grant : Healthy Youth/Subvention catalyseur : Jeunes en santé
**Title/Titre:**	Identifying health research and knowledge mobilization priorities for promoting health equity in Canada's youth

Assessment/Évaluation:

Would like to have seen more evidence of feasibility of the approach. The team has experience, but specific description for this project would have been helpful.

The issue is presented as a broad and general health equity issue in Canada, but the dissemination of the information to be generated seemed sparce. If the goal is to create a research agenda, I would have expected to see more ideas around next steps.

The applicants state “The proposed project is well positioned to advance youth health equity in Canada through identifying and prioritizing knowledge mobilization and research foci that will advance equity-oriented health services, health systems change, and long-term sustained action toward improving young people’s health outcomes.” This is probably an overstatement – how will all this occur based on the findings of this 1 year project?

Environment

Excellent

Sex and gender

Embedded into their design, data collection, and interpretation. Very thoughtful, although lacking in details at times.

Budget is low – travel costs for a conference at $1000 per person???
