# Supplementary material for: Health Research and Knowledge Mobilization Priorities for Youth Health Equity in Canada: Protocol for a Multi-Methods Study
Source: JMIR Res Protoc. 2026 Sep 16;15:e84782. doi: 10.2196/84782 (PMC13628041; doi:10.2196/84782)
Supplement: Supplementary file 4 [file resprot_v15i1e84782_app4.pdf]

**Review Type/Type d'évaluation:**	Committee Member 3/Membre de comité 3
**Name of Applicant/Nom du chercheur:**	Hilario, Carla Theresa
**Application No./Numéro de demande:**	513361
**Agency/Agence:**	CIHR/IRSC
**Competition/Concours:**	2023-11-08 Catalyst Grant: Healthy Youth/Subvention catalyseur : Jeunes en santé
**Committee/Comité:**	Catalyst Grant : Healthy Youth/Subvention catalyseur : Jeunes en santé
**Title/Titre:**	Identifying health research and knowledge mobilization priorities for promoting health equity in Canada's youth

Assessment/Évaluation:

Summary

The proposed research aims to better understand knowledge mobilization for promoting health equity for youth and how this can be turned into action. The proposed study will examine the perspectives of equity seeking youth across Canada and priority areas for future research related to health equity using a series of focus groups and virtual sessions with youth.

Evaluation Criteria

Research Approach

The research approach appears to be very youth engaged and even led by youth throughout. The methodology is comprehensive and makes the impact of the research for youth clear. More details about the recruitment of diverse youth could have been included to better understand how sample size and retention of participants would be achieved.

Originality

The proposed research is very original in nature, taking a new and comprehensive approach to better understanding how we can best translate knowledge into action to address health inequity in Canada for youth. The project would make a significant and novel contribution to the area of study.

Applicants

The research team is mainly composed of early career researchers and the proposal does a great job of including their multiple youth voice representatives as research team members. Further, their youth representative reviewers are also knowledge users and do not overlap with the principal researchers.

Environment

Details about the research environment, space for research team, and students does not appear to be provided. The focus groups with youth appear to be virtual, therefore physical space does not seem to be needed for that part of the research plan.

Impact

The proposed project will make a significant contribution to knowledge translation as it is central to its concept, however more specific details about how results would be translated and made accessible to youth themselves could have been made clearer. Much of the knowledge translation appears to be geared towards other researchers and the general public.

Sex/Gender

The proposed research does a good job differentiating between sex and gender and describes how these factors will be considered in their research. The research project is also focused on having an inclusive and diverse representation of youth and clearly outlines the important of gender diverse youth.

**Review Type/Type d'évaluation:**	Committee Member 3/Membre de comité 3
**Name of Applicant/Nom du chercheur:**	Hilario, Carla Theresa
**Application No./Numéro de demande:**	513361
**Agency/Agence:**	CIHR/IRSC
**Competition/Concours:**	2023-11-08 Catalyst Grant: Healthy Youth/Subvention catalyseur : Jeunes en santé
**Committee/Comité:**	Catalyst Grant : Healthy Youth/Subvention catalyseur : Jeunes en santé
**Title/Titre:**	Identifying health research and knowledge mobilization priorities for promoting health equity in Canada's youth

Assessment/Évaluation:

Budget

The budget appears to be appropriate and reasonable. The proposal does a great job of the majority of their budget to include and support student researchers on the team. The budget also includes a small Honoria for all research participants.
